# Casimersen (AMONDYS 45™): An Antisense Oligonucleotide for Duchenne Muscular Dystrophy

**DOI:** 10.3390/biomedicines12040912

**Published:** 2024-04-20

**Authors:** Milyard Assefa, Addison Gepfert, Meesam Zaheer, Julia M. Hum, Brian W. Skinner

**Affiliations:** 1 School of Medicine, University of Virginia, Charlottesville, VA 22903, USA; mma5df@virginia.edu; 2 College of Osteopathic Medicine, Marian University, Indianapolis, IN 46222, USA; agepfert067@marian.edu (A.G.); mzaheer215@marian.edu (M.Z.); 3 Division of Biomedical Sciences, College of Osteopathic Medicine, Marian University, Indianapolis, IN 46222, USA; 4 Division of Clinical Sciences, College of Osteopathic Medicine, Marian University, Indianapolis, IN 46222, USA; bskinner@marian.edu

**Keywords:** AMONDYS 45, antisense oligonucleotide, casimersen, Duchenne muscular dystrophy, dystrophin, exon skipping, neuromuscular disorders, phosphorodiamidate oligomer

## Abstract

Casimersen (AMONDYS 45^TM^) is an antisense oligonucleotide of the phosphorodiamidate morpholino oligomer subclass developed by Sarepta therapeutics. It was approved by the Food and Drug Administration (FDA) in February 2021 to treat Duchenne muscular dystrophy (DMD) in patients whose *DMD* gene mutation is amenable to exon 45 skipping. Administered intravenously, casimersen binds to the pre-mRNA of the *DMD* gene to skip a mutated region of an exon, thereby producing an internally truncated yet functional dystrophin protein in DMD patients. This is essential in maintaining the structure of a myocyte membrane. While casimersen is currently continuing in phase III of clinical trials in various countries, it was granted approval by the FDA under the accelerated approval program due to its observed increase in dystrophin production. This article discusses the pathophysiology of DMD, summarizes available treatments thus far, and provides a full drug review of casimersen (AMONDYS 45^TM^).

## 1. Introduction

Duchenne muscular dystrophy (DMD) is an X-linked recessive disorder that causes a degenerative neuromuscular disease that significantly affects the skeletal and cardiac muscles, resulting in debilitating muscle weakness. The consequences of this condition extend beyond muscle impairment, leading to decreased heart function and a weakened diaphragm that can lead to respiratory and/or cardiac failure [1,2]. This disorder is one of the most severe and the most common inherited forms of childhood muscular dystrophy, imposing a heavy burden on those affected [1]. Like any other X-linked recessive disease, it occurs most frequently in males, affecting approximately 1 in 3000–5000 live male births [3]. While it can occur in females, it is very rare, affecting 1 in 50,000,000 live female births [4]. Even when females inherit the abnormal X chromosome, they are usually heterozygous asymptomatic carriers. Very rarely can a female present with signs and symptoms of DMD due to homozygous mutations in the dystrophin gene. DMD does not exhibit a predilection for any race or ethnic group [5]. According to one study, two-thirds of DMD cases are due to mutations on the X chromosome from a female carrier while the remaining cases are due to de novo mutations [2].

DMD is caused by mutations of the *DMD* gene, the largest gene in the human genome, which codes for a protein that is essential for the structure of a myocyte membrane (DP427m) [2]. Mutations of the *DMD* gene could either be frameshift or point mutations that prevent the synthesis of a fully functional dystrophin protein by truncating the sequence. Several animal models exist to explore potential gene therapies for DMD, with muscular dystrophy X-linked (mdx) mice being the most common model used for these studies [6].

One recent study using mdx mice utilized clustered regularly interspaced short palindromic repeats/CRISPR-associated 9 (CRISPR/Cas9) gene editing in combination with a homology-directed repair (HDR) donor vector to replace a mutated exon, exon23, to restore dystrophin expression in stem cells for transplantation [7]. However, the efficacy of these therapies is still limited by the low survival rate of dystrophin-expressing donor cells.

Beyond mouse models, certain investigations utilize canine DMD models [8]. In a recent study, dual adeno-associated virus (AAV) technology was utilized alongside the mini-dystrophin gene, a truncated version of the dystrophin gene, to restore components of the dystrophin-associated glycoprotein complex (DGC) on the sarcolemma [8]. Nonetheless, these therapeutic approaches faced limitations as the study involved only one mixed-breed dog, comprising golden retriever, labrador retriever, and beagle ancestry. Moreover, the treatment duration spanned only 2 months, raising doubts regarding its long-term effectiveness.

The earliest symptoms can be seen around 2–3 years of age and often include difficulty in climbing stairs, a waddling gait, and frequent falls [9]. Untreated, DMD worsens quickly and is characterized by muscle wasting and loss of walking ability, leading to complete wheelchair dependence on average by approximately age 13 [10]. While cardiovascular symptoms are rare before the age of 20, cardiac impairment can occur around 6–10 years of age [2]. With optimal care, most DMD patients die between 20 and 40 years of age from cardiac and/or respiratory issues [9].

DMD is one of the nine types of muscular dystrophy. Another type of muscular dystrophy that presents as a milder form of DMD is Becker muscular dystrophy (BMD) [11]. Similar to DMD, BMD is also an X-linked recessive disorder caused by a mutation to the dystrophin gene, leading to progressive muscle degeneration and proximal muscle weakness [12]. Due to its inheritance pattern, BMD also primarily affects young males. However, the mutations in BMD affect a much smaller part of the dystrophin gene than that of DMD. Studies have found that immunohistoanalysis on DMD patients shows total absence of dystrophin, whereas analysis of BMD patients shows 10–40% of the normal amount of dystrophin [13]. Hence, BMD has a less severe disease course and a more gradual progression. The age of onset for BMD is later than DMD, with symptoms usually appearing after the age of 8 [14]. While DMD patients are usually dependent on wheelchairs by age 13, BMD patients usually remain ambulant until the age of 16 [13]. BMD also has a lower prevalence than DMD, with some studies showing BMD to be three times less common than DMD in the United States [15]. This review outlines the pathophysiology of DMD, the history of antisense oligonucleotide (ASO) therapy, and the pharmacology of casimersen.

## 2. Pathophysiology

**Figure 1:**
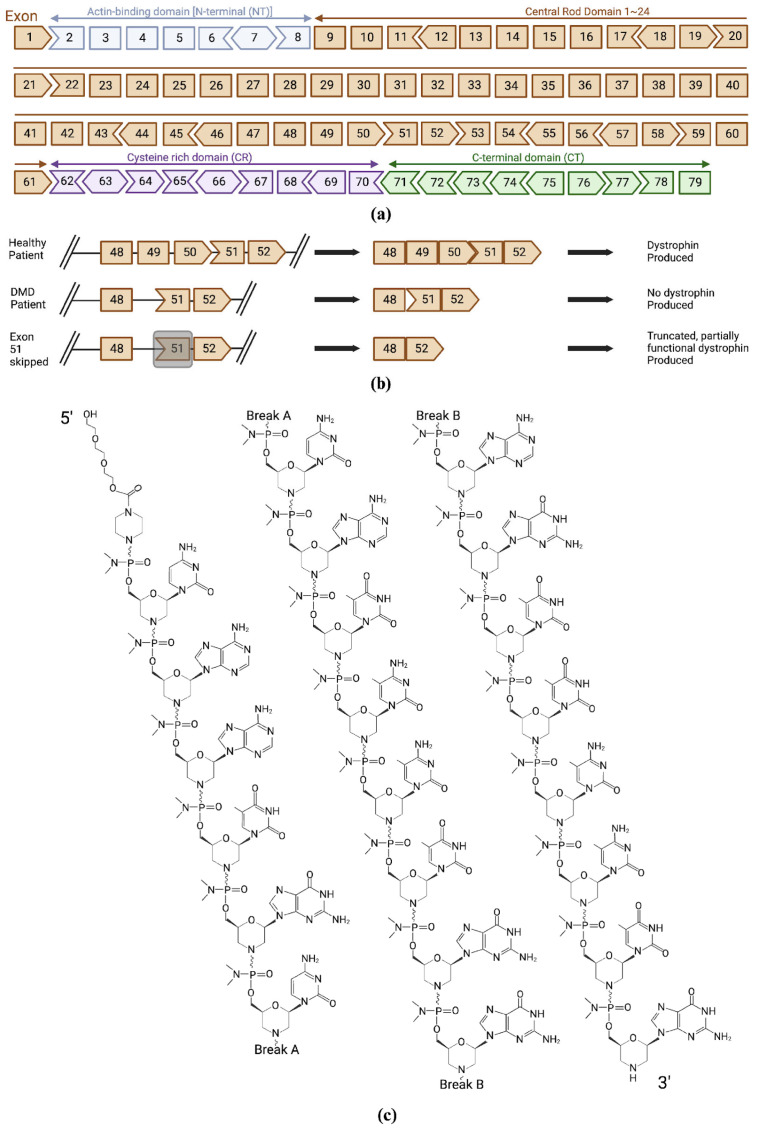
(**a**) The structure of the dystrophin gene is shown with all 79 exons and corresponding domains: N terminus (Actin binding), central rod domain, cysteine-rich domain, and C-terminal domain. (**b**) Antisense-induced exon skipping in DMD patients. Deletion of exons 49 and 50 resulted in premature stop codon and disrupted the production of dystrophin. Exon skipping of 51 in this case produced a truncated and partly functional dystrophin protein. (**c**) Structure of casimersen.

Dystrophin is a membrane-associated protein of 3685 amino acids with a molecular weight of 427 kDa [Figure 1a]. Dystrophin has four domains: an N-terminal domain for binding actin, a central rod-like domain mainly for structural flexibility, a cysteine-rich domain for facilitating protein–protein interactions and stabilizing dystroglycan binding, and a C-terminal domain for binding several cytoplasmic, integral membranes and dystrophin-associated protein complexes (DAPC) at the sarcolemma [2]. It links cytoskeletal actin muscle fibers with the extracellular matrix. This linkage strengthens muscle structure during stressful contraction/relaxation cycles. The loss of dystrophin protein predisposes muscle fibers to mechanical damage, leading to muscle degeneration and cardiomyopathy [9].

Muscle degeneration due to loss of dystrophin activates the innate immune system as part of the normal repair process. While acutely this facilitates the repair process, the release of cytokines, neutrophils, and macrophages chronically in DMD patients due to cellular stress leads to the replacement of muscle tissue with fibrous tissue. In the heart, replacement with fibrous tissue generally affects the posterior, lateral, and segments of the anterior portions of the ventricles, leading to ventricular dilation and dysfunction [2]. Thus, targeting the immune system to reduce the inflammatory response in DMD patients has been of interest and one of the mainstays in their therapy.

## 3. Treatment for Duchenne Muscular Dystrophy

DMD is currently incurable; thus, prolongation of walking and cardiac and respiratory function has been the major aim of treatment. Among the standard treatments for DMD is the use of glucocorticosteroids. Several randomized controlled trials (RCTs) have demonstrated that corticosteroids significantly improve muscle strength and function in boys with DMD in the short term due to their anti-inflammatory nature [16]. However, questions regarding their long-term adverse effects like excessive weight gain, bone weakness, and muscle atrophy have made corticosteroids less preferable options. Recently several studies have been conducted to better understand corticosteroid’s mechanism of action in the muscle repair process in order to maximize therapeutic options for DMD patients while minimizing their adverse effects [17]. In this light, vamorolone was recently approved by the FDA in October 2023 as a potential treatment for patients with DMD above 2 years of age [18]. Vamorolone is a steroid analogue with a distinct chemical structure that works by inhibiting inflammatory pathways, thereby promoting cell membrane stability. It acts as an inhibitor to the mineralocorticoid receptor, thereby decreasing some of the adverse effects like hypertension and bone weakness compared to corticosteroids. Unlike corticosteroids, vamorolone does not suppress the immune system or bone growth, making it a favorable option. However, cushingoid features and other unknown potential long-term adverse effects still remain of concern. Thus, although steroids and vamorolone remain as part of the recommendation to treat DMD, several questions remain unanswered regarding their long-term consequences and efficacy in prolonging walking [18].

Several other approaches have also been considered to treat DMD besides anti-inflammatory agents like corticosteroids and vamorolone. One approach is the development of therapies by targeting the restoration of the production of the functional dystrophin protein. This approach involves using antisense oligonucleotides which bind to target exons in the pre-mRNA dystrophin transcript to skip early stop codons in the dystrophin gene and to restore the reading frame, leading to the production of truncated but functional dystrophin protein [1].

Due to mutations that can happen in various exon regions coding for the protein, DMD can express itself in various ways [Figure 1b]. Depending on the region of mutation, there can be different forms of DMD. For example, one form of DMD is a mutation in the exon 51 region of the sequence which accounts for about 14% of DMD patients. In September 2016, the Food and Drug Administration (FDA) granted accelerated approval for eteplirsen (EXONDYS 51^TM^) to treat DMD patients with a mutation that is specific to the region exon 51. Eteplirsen is a phosphorodiamidate morpholino (PMO) antisense oligonucleotide (ASO) drug that acts to promote dystrophin production by restoring the translational reading frame of DMD by skipping exon 51 in defective gene variants [3]. Common adverse effects include headache, fever, nausea, and abdominal pain [19].

Another form of DMD stems from a mutation in the exon 53 region, which accounts for about 8% of DMD patients [20]. Other exon-skipping therapies, such as golodirsen and viltolarsen, have been developed to treat this form of DMD. 

Golodirsen (Vyondys 53), like eteplirsen, is a PMO ASO drug that was approved by the FDA in 2019 for the treatment of DMD [21]. Golodirsen works by skipping exon 53 to create a truncated, but functional, dystrophin gene [22]. It is important to note that this therapeutic approach is not a cure. Golodirsen can slow the progression of DMD, but it cannot revive muscle tissue that has already been lost [21]. Common adverse effects include rash, fever, headache, itching, and abdominal pain [23].

Viltolarsen (Viltepso) is an ASO drug that was granted accelerated approval by the FDA in 2020 to treat a specific type of DMD [24]. Its mechanism is similar to that of golodirsen, in that it skips exon 53, increasing the amount of dystrophin. However, viltolarsen is the first of these exon 53-skipping therapies to show an elevation in dystrophin levels in children as young as 4 years old [25]. Common adverse effects include injection-site reactions, cough, headache, arthralgias, and urticaria [26].

While the ASO drugs mentioned above treat DMD by targeting either exon 51 or exon 53, another form of DMD is expressed as a result of a mutation occurring in the exon 45 region. This DMD type consists of approximately 8% of all DMD patients. In February 2021, the FDA approved casimersen (AMONDYS 45™), a drug that has been approved to target a translation reading frame of DMD in the exon 45 region.

## 4. Other Antisense Oligonucleotides Brought to Market

ASO drugs are single-stranded nucleic acid polymers primarily used to treat common and rare human diseases. They function by binding to a specific portion of an mRNA sequence involved in the formation of a specific disease, thereby interfering with its gene expression [27]. Mechanisms of interference include inhibiting translation, promoting RNA degradation, and modulating RNA splicing [28]. ASO drugs that induce RNA degradation utilize the binding of ASO to mRNA sequence results in the activation of RNase, an enzyme that cleaves and degrades RNA. Genes that cause or modify diseases are common targets of RNaseH1-mediated ASOs [29]. Another common mechanism implored by ASO drugs is inhibition of translation via splicing modulation. Splicing modulation can be used to induce exon skipping or exon inclusion to restore a functional reading frame. Drugs that induce alternative splicing can be used to treat diseases caused by frameshift mutations [29].

These drugs have been around for decades, with fomivirsen (Vitravene) being the first FDA-approved ASO drug back in 1998 for the treatment of cytomegalovirus (CMV) retinitis [30]. Human CMV is a ubiquitous herpes virus that is the most common cause of viral retinitis in immunocompromised individuals, such as HIV patients [31]. This ASO drug works by specifically binding the viral mRNA to inhibit the replication of human CMV [32]. It is administered intravitreally, and common adverse effects include abnormal or blurred vision, changes in color vision, eye pain, eye redness, and eye sensitivity to light [33]. Due to the efficacy of antiretroviral therapies for the treatment of CMV retinitis, Fomivirsen was removed from the market in 2002 and 2006 in the European Union and US, respectively [34].

After fomivirsen, pegaptanib (Macugen) was approved by the FDA in 2004 for the treatment of neovascular age-related macular degeneration [35]. Pegaptanib is an aptamer, which is an oligonucleotide ligand that binds to selective molecular targets. Pegaptanib specifically is an RNA aptamer that is chemically modified and attached to polyethylene glycol (PEG). This modification provides structural stability and improves the pharmacokinetics of pegaptanib. Pegaptanib targets vascular endothelial growth factor (VEGF)-165. VEGF is essential for the growth of vascular endothelial cells and the formation of new blood vessels [36]. However, in age-related macular degeneration, these abnormal blood vessels leak fluid, damaging the macula and central vision. VEGF-165 is the specific isoform implicated in the ocular neovascularization process. Pegaptanib is administered intraocularly, and common adverse effects include bladder pain, blurred vision, eye pain, sore eyes, and tunnel vision [37]. Pegaptanib is credited with being the first FDA-approved aptamer, laying the foundation for future aptamer therapies.

Mipomersen (Kynamro) was approved by the FDA in January 2013 for the treatment of familial hypercholesterolemia [38]. This ASO drug inhibits apolipoprotein B (apo-B), which plays a crucial role in lipid metabolism. Apolipoprotein B is essential for the function of very low-density lipoprotein (VLDL) and low-density lipoprotein (LDL). VLDL is synthesized by the liver and transports cholesterol and triglycerides to peripheral tissues [39]. By altering this function of VLDL, mipomersen contributes to fat accumulation in the liver, causing non-alcoholic steatohepatitis (NASH), which can progress to liver cirrhosis. Common adverse effects include injection-site reactions, such as pain, erythema, and pruritus, along with flu-like symptoms [40]. 

Nusinersen (Spinraza) was approved by the FDA in 2016 for the treatment of spinal muscular atrophy. Spinal muscular atrophy is caused by a gene mutation in the Survival Motor Neuron (SMN) 1 gene. A shorter, truncated protein is produced by the *SMN2* gene, though it cannot compensate for the loss of *SMN1*. Spinraza targets the *SMN2* gene, which then promotes full length SMN proteins lost by the mutation of the *SMN1* gene [41]. Common adverse effects include lower respiratory infection, fever, constipation, headache, and vomiting [42].

Defibrotide sodium (Defitelio) is a deoxyribonucleic acid derivative that was approved by the FDA in 2016 for the treatment of hepatic veno-occlusive disease (VOD), also known as sinusoidal obstruction syndrome (SOS) [43]. VOD occurs in patients that have undergone hematopoietic stem-cell transplantation [44]. Defibrotide sodium works to prevent blood clot formation by selectively increasing prostaglandin I2 and E2 levels, promoting tissue plasminogen activator, and decreasing the activity of tissue plasminogen inhibitor [45]. This enhanced fibrinolytic activity helps restore sinusoidal blood flow. Common adverse effects include hypotension, diarrhea, vomiting, nausea, and epistaxis.

Inotersen (Tegsedi) is an ASO drug that was approved by the FDA in 2018 for the treatment of hereditary transthyretin amyloidosis (ATTR) [46]. ATTR is caused by mutations in the transthyretin (TTR) gene, which leads to a buildup of a misfolded protein called amyloid [47]. This amyloid is deposited in the body’s organs and tissues, which can lead to cardiomyopathy and the dysfunction of other organs [48]. Inotersen works by binding to mRNA from the TTR gene and preventing its translation [49]. Common adverse effects include injection-site reactions, nausea, headache, tiredness, low platelet counts, and fever [46]. 

## 5. Concerns with the Use of Antisense Oligonucleotides

While many of the aforementioned drugs have had success, many have not made it to market or have been pulled from market due to hepatotoxicity concerns. Many studies have evaluated possible causes for the hepatotoxicity seen across the various ASO drugs, despite the lack of common treatment targets. One study noted that the most hepatotoxicity was seen in ASOs that utilized bicylic modifications (BNA) like locked nucleic acid (LNA) to induce cleavage of the intended RNA target. The study also showed that ASO drugs with the most hepatotoxicity, measured by liver function test (transaminases) elevations, were intended to downregulate transcripts with long pre-mRNA sequences. The more severe the hepatotoxicity, the larger the magnitude of downregulation and the longer the length of the mRNA transcripts [50]. ASOs utilizing LNA modification have been previously shown to cause hepatotoxicity independent of the target mRNA sequence and modulation [51]. As most of the documentation regarding ASOs’ hepatotoxicity is in reference to LNA modifications, it is unclear whether the hepatotoxicity is specific to LNA modifications or to high-affinity modifications in general. LNA modifications were substituted with 2′-O-methoxyethyl (MOE) and constrained ethyl (cEt) to test this hypothesis. There was complete suppression of the alanine aminotransferase (ALT) increase in those with 2′-MOE; however, there was a reduced potency regarding on-target transcripts. Substitutions with cEt showed moderate suppression of the ALT increase while not affecting the potency of on- or off-target transcripts. 

Additionally, a study assessed the methods in which the downregulation of the gene expression was induced. Many ASO drugs utilize ribonuclease H (RNase H) and small interfering RNA (siRNA) to promote DNA degradation, and the commonality of the downregulation suggests that RNase H1 is likely involved. To identify if RNase H1 contributes to the downregulation seen, mice were pre-treated with an anti-RNase H1 MOE ASO or a control oligonucleotide, then treated with a toxic LNA ASO. Pre-treatment with anti-RNase H1 exhibited a reduction in liver enzymes and no evidence of liver injury. Conversely, pretreatment with a control oligonucleotide exhibited no reduction in liver enzymes, while also displaying no evidence of liver injury. These findings suggest that the downregulation of transcripts is RNase H1-dependent. The combination of hepatotoxicity seen in ASO drugs utilizing LNA modifications and cEt modifications, as well as the severity of the hepatotoxicity dependent on the length of the pre-mRNA and the downregulation of expression, suggests that hepatotoxicity likely results from incorporating high-affinity modifications and is not solely from LNA mechanisms [49].

Studies have also looked at the efficacy and delivery of ASO drugs, as nucleic-acid-based therapies have historically had low bioavailability and uptake. Drugs utilizing antisense biomolecules are often highly charged and have a high molecular weight, making it difficult for them to cross the cell membrane. Cell-penetrating peptides (CPPs) have been shown to help increase bioavailability through two methods: through direct attachment to the drug or through noncovalent nanoparticle complexes to increase cellular uptake. Direct attachment, or covalent conjugation, typically occurs between a charge-neutral oligonucleotide and a cationic CCP. The addition of the CCP improves cellular uptake and enhances antisense activity. Covalent CCPs represent a highly reproducible, though laborious compound with limited oligonucleotide targets. Cationic CCPs are limited when conjugated with negatively charged nucleic acid oligomers, as there is an electrostatic interaction that can potentially interfere with the target binding site. Increasing bioavailability and uptake in anionic oligonucleotides is best achieved using noncovalent CCP methods. Noncovalent CCPs utilize the formation of nanoparticle complexes from electrostatic or hydrostatic interactions between the anionic oligonucleotide and an amphipathic peptide. Limitations to noncovalent CCPs include increased potential for degradation by intracellular and extracellular proteases. Noncovalent CCPs represent more simplified interactions between particles and targets with a wider usage, but a less reliable target compound [52]. 

Current research is now focusing on ways to assess the risk of hepatotoxicity in earlier phases of drug development. As studies have shown that the least selective ASOs exhibit the most hepatotoxicity, utilizing transcriptome-wide screening for selectivity is an initial step to assess hepatotoxicity in the drug development stage [53]. Additional research remains to be conducted on CCP delivery and other methods to enhance the bioavailability and cellular uptake of ASOs [52]. Given the success of ASO drugs for other therapies, casimersen represents a novel treatment option for the management of DMD. This paper focuses on the pharmacology of casimersen, including its use, adverse effects, and considerations prior to initiation of therapy.

## 6. Description of Casimersen

Casimersen is a subclass of PMO. PMOs are synthetic molecules in which a five-membered ribofuranosyl ring in DNA and RNA are replaced by a six-membered morpholino ring linked through uncharged phosphorodiamidate moieties. Casimersen has a molecular formula of C_268_H_424_N_124_O_95_P_22_ and weighs 7584.5 daltons. It contains 22 linked subunits with a sequence of bases of 5′-CAATGCCATCCTGGAGTTCCTG-3′ [Figure 1c]. It is available as a concentrated 50 mg/mL aqueous solution designed for intravenous infusion post-dilution. The final formulation comprises 50 mg casimersen, 0.2 mg potassium chloride, 0.2 mg potassium phosphate monobasic, 8 mg sodium chloride, and 1.14 mg sodium phosphate dibasic. [54].

## 7. Clinical Pharmacology of Casimersen

### 7.1. Mechanism of Action

Casimersen is an antisense oligonucleotide indicated for the treatment of DMD in patients who have mutations in the exon 45 region of the dystrophin gene. It works by binding to the mutated portion of the dystrophin pre-mRNA, specifically exon 45. Upon binding, casimersen induces splicing of the mutated exon, thereby allowing the production of functional dystrophin protein. Although truncated, the resulting dystrophin gene remains functional. Through this mechanism, casimersen slows the progression of muscle disease in patients with DMD [1].

### 7.2. Pharmacodynamics

During the ESSENCE trial (NCT02530905), an interim analysis was conducted at week 48 by obtaining a muscle biopsy to determine the efficacy of casimersen. Patients were divided into two groups where one group received casimersen and the other received a placebo. Patients who received casimersen (*n* = 27) showed a significant increase in the skipping of exon 45 (*p* < 0.001) compared to baseline. On the other hand, compared to baseline, patients who received placebo (*n* = 16) did not demonstrate a significant increase in exon 45 skipping (*p* = 0.808). Exon 45 skipping and dystrophin protein expression have a positive correlation, indicating casimersen as a potential drug to treat exon 45-amenable DMD [55].

In the ESSENCE trial, dystrophin level was also assessed by Western blot analysis, which showed an increase from 0.93% (SD 1.67) at baseline to 1.74 (SD 1.97) after 48 weeks of treatment with casimersen. The study also used immunofluorescence staining to demonstrate correct localization of dystrophin protein in the sarcolemma [54].

### 7.3. Pharmacokinetics

DMD patients were administered IV doses of 4 mg/kg/week–30 mg/kg/week of casimersen to evaluate pharmacokinetic properties. At the end of a single IV dose, Cmax was achieved [54]. Over a 24 h period, plasma concentrations of casimersen, across all doses, rose and declined in a similar manner. Long-term weekly dosing did not result in an accumulation when comparing week 7 to week 60 [56].

### 7.4. Distribution

Casimersen undergoes non-concentration-dependent distribution with plasma protein binding, ranging from 8.4% to 31.6%. Following a 30 mg/kg dose, casimersen had a steady-state volume of distribution of 367 mL/kg, with a variability of 28.9% [54].

### 7.5. Metabolism

Casimersen does not undergo extensive hepatic metabolism, and no known metabolites are detectable in plasma or urine [54].

### 7.6. Elimination and Excretion

Plasma clearance of casimersen was 180 mL/h/kg at 30 mg/kg dose and has an elimination half-life of 3.5 h. Casimersen is excreted mostly in urine and is unchanged. According to the FDA, a clinical study showed more than 90% of casimersen to be excreted in urine, with negligible fecal excretion [54]. Renal clearance is suggested to be the main route of elimination, as total clearance from renal excretion ranged from 77% to 108% [56]. 

### 7.7. Handling and Storage

Casimersen should be stored below room temperature to maintain its integrity. It should never be stored in the freezer, as extreme temperatures alter its pharmacodynamics. The ideal storage temperature for casimersen is 2 °C to 8 °C (36 °F to 46 °F). 

### 7.8. Administration, Dosage, and Strengths 

Casimersen is administered intravenously after dilution with normal saline to a total volume of 100 to 150 mL. Prior to infusion, the line should be flushed with 0.9% NaCl. It should not be administered with or mixed with other medications [54]. The dose recommended for administration of casimersen is 30 mg/kg/week as a 35 to 60 min intravenous infusion via an in-line 0.2-micron filter. If a dose is missed, it can be administered as soon as possible after the missed dose [54].

### 7.9. Use in Specific Populations

There are no human or animal data available to assess the effect of administering casimersen to pregnant women. Since DMD is largely a disease affecting children and young adults, there is no evidence of its use in and effect on geriatric patients. The efficacy and adverse effects of casimersen have been assessed on children and young adults with a specific mutation amenable to exon 45 skipping. 

An animal study that assessed the safety of intravenous casimersen indicated that high doses of casimersen cause renal tubular damage [55]. In this study, casimersen was administered intravenously at varying concentrations (0, 100, 300, and 900 mg/kg) to juvenile rats once a week for 10 weeks. Renal tubular degeneration was observed at the highest dose, but no effects were observed on the reproductive system, development, and immune function [54]. Similarly, studies showed a transient nephrotoxic effect when casimersen was administered to cynomolgus monkeys at a dose > 40 mg/kg. However, this effect resolved weeks later after the last dose [57].

### 7.10. Adverse Effects and Contraindications

#### Clinical Trial

In a double-blind, placebo-controlled study, 76 patients with a demographic of age between 7 and 20 years, of white (88%) and Asian (9%) ethnicities, and confirmed to have DMD were enrolled. In the clinical trial, a dose of at least 30 mg/kg casimersen was administered once a week. In the study, patients either received casimersen (*n* = 57) or placebo (*n* = 31) intravenously once a week for up to 96 weeks. After the 96 weeks, all patients received 30 mg/kg casimersen for 48 weeks. Adverse reactions were 20% greater in patients that received casimersen than in the placebo group. Those that occurred in 20% of casimersen-treated patients and 5% of placebo patients can be seen in Table 1 [55].

An additional randomized, double-blind, placebo-controlled, dose-titration trial was conducted on 12 participants aged 7–21 years. Participants underwent a 12-week dose titration where they were randomized 2:1 to weekly casimersen infusions or placebo. The casimersen infusions were titrated using escalating doses of 4, 10, 20, and 30 mg/kg, and patients spent two weeks at each dose. Adverse reactions were classified as treatment-emergent (TEAEs) if they developed, worsened, or became serious at the start of the infusions or within 28 days after the last dose. Serious adverse reactions (SAEs) were those which were life-threatening, required hospitalization, or caused significant disability. Over the 12-week period, all patients experienced one TEAE, with those related to treatment reported at the same frequency between the treatment (2 out of 8, 25%) and the placebo (1 out of 4, 25%) groups. Those in the treatment group were mild and considered unrelated to the drug. During the double-blind treatment, the most commonly reported TEAEs were pain with the procedure, headache, and vomiting. The most commonly reported TEAEs during the combined double-blind and open-label period were nasopharyngitis, cough, headache, procedural pain, upper respiratory tract infection, and vomiting [56].

### 7.11. Warnings and Precautions

Kidney function should be monitored in patients that receive casimersen. Although not observed in human studies of casimersen, kidney toxicity and glomerulonephritis have been noted in animals who received casimersen, particularly at supratherapeutic doses. It is recommended to measure glomerular filtration rate before administering casimersen and to continue monitoring kidney function using urine dipstick, serum cystatin C, and urine protein creatinine ratio (UPCR) every three months [58]. 

Given the concerns raised about ASO-mediated hepatotoxicity, serum transaminase levels were not elevated in clinical trials, and there have been no reported episodes of liver injury clinically seen [59]. Casimersen utilizes PMOs to alter the splicing site, inducing exon skipping to produce functional protein synthesis [29]. Studies have shown that most hepatotoxicity is likely related to the high-affinity modifications used in ASOs and to the downregulation of the target mRNA via RNaseH1 mechanisms [50]. Studies testing PMO drugs, including casimersen, have not seen the systemic toxicities and target organ toxicities associated with other ASO drugs [57].

## 8. Efficacy of Casimersen

Interim results of the ongoing phase III ESSENCE trial (NCT02530905) have shown that casimersen increases dystrophin production in patients with DMD amenable to exon 45 skipping [60]. 

The ESSENCE trial included male patients in the age range of 6–13 who can ambulate (defined by a mean 6 min walk test of ≥300 m and ≤450 m), have stable pulmonary function (percent predicted forced vital capacity greater than 50%), and who were on a stable corticosteroid dose for a duration of 6 months or greater prior to the start of the trial [1]. On the other hand, patients on other DMD treatments, undergoing treatment with gene therapy, clinically significant illness, or major surgery within the past 3 months were excluded from the trial [1,55,56]. Eligible participants underwent a double-blind 12-week dose titration where they were randomized 2:1 to weekly casimersen infusions or placebo. The casimersen infusions were titrated using escalating doses of 4, 10, 20, and 30 mg/kg, and patients spent two weeks at each dose [55]. An open-label extension period lasting 132 weeks followed, where all 12 initial participants were enrolled and received casimersen. Twelve males meeting the criteria participated in total, with eight assigned to the casimersen group and the remaining four assigned to the placebo during the double-blind portion of the trial. Of the initial 12, 11 completed the open-label extension period. The primary end point was a 6 min walk test change from baseline at 96 weeks. The secondary end point for the trial was a 6 min walk test change from baseline at week 144, dystrophin protein change from baseline at weeks 48 and 96, change in forced vital capacity from baseline at weeks 96 and 144, as well as other ambulation assessments like the ability to rise from a seated position independently and time to loss of ambulation assessed by the North Star ambulatory assessment score [1,55,56]. 

Although still ongoing, the interim results from this trial via Western blot analysis showed increased mean dystrophin levels, which was measured as a percentage of normal. It increased from 0.93% normal at baseline to 1.74% normal at week 48 among patients treated with IV casimersen 30 mg/kg once weekly (mean change from baseline, 0.81; *p* < 0.001) compared with a change from 0.54% normal to 0.76% normal among placebo recipients (mean change from baseline, 0.22; *p* = 0.09) [1,56]. The mean change from baseline shows a between-group difference of 0.59 (*p* = 0.004) [56]. All casimersen recipients displayed an increase in exon 45 skipping (100% response rate). Furthermore, a positive correlation between exon 45 skipping and dystrophin production was noted (Spearman rank correlation, 0.627; *p* < 0.001) [1].

## 9. Conclusions

Casimersen is an antisense oligonucleotide developed by Serepta therapeutics and conditionally approved by the FDA as the first line treatment for patients with DMD amenable to mutation on exon 45. Conditional approval by the FDA is granted if the immediate availability of a drug fulfills an unmet medical need, and if the benefit outweighs the risks. Currently, the clinical trial for casimersen is ongoing and expected to be concluded in the second quarter of 2024. It requires a full approval and verification of clinical benefit in a phase 3 ESSENCE study (NCT02500381) [61]. An additional extension study (NCT03532542) with estimated enrollment of 260 participants is expected to be completed in 2026 [62]. Clinically, the administration of casimersen to patients with mutation in the exon 45 region has shown a statistically significant production of dystrophin protein in skeletal muscles compared to the placebo group.

### What Does This Mean for DMD Patients and What Is Next?

The approval and clinical use of casimersen is expected to treat about 8% of all DMD patients. Currently, along with other RNA therapies, a treatment plan can be provided for nearly 30% of DMD patients. While there is a lot of progress to be made in this area and casimersen still requires full approval from the FDA, the discovery of the drug is an invaluable addition to the treatment of DMD and provides hope for DMD patients. Additionally, besides ASO therapies, which are the main focus of this paper, trials for other treatment options like the monoclonal antibody pamrevlumab, synthetic steroids like vamorolone, and other allogeneic stem cell therapies are underway. With all these recent discoveries and undergoing trials, even if a complete cure might be difficult to attain, we hope that significant strides can be made to treat more DMD patients in the next decade. 

## Figures and Tables

**Table 1:** Adverse reactions of casimersen occurring in at least 20% of patients.

Adverse Reactions	AMONDYS 45 30 mg/kg Once Weekly (*n* = 57) %	Placebo (*n* = 31) %
Upper Respiratory Tract Infections	65	55
Cough	33	36
Pyrexia	33	23
Headache	32	19
Arthralgia	21	10
Oropharyngeal Pain	21	7

## References

[1] Shirley M. (2021). Casimersen: First Approval. Drugs.

[2] Adorisio R., Mencarelli E., Cantarutti N., Grandinetti M., Adorisio R., Mencarelli E., Cantarutti N., Grandinetti M. (2021). Cardiomyopathy in Duchenne Muscular Distrophy: Clinical Insights and Therapeutic Implications. Cardiomyopathy—Disease of the Heart Muscle.

[3] Lim K.R.Q., Maruyama R., Yokota T. (2017). Eteplirsen in the Treatment of Duchenne Muscular Dystrophy. Drug Des. Devel. Ther..

[4] Nozoe K.T., Akamine R.T., Mazzotti D.R., Polesel D.N., Grossklauss L.F., Tufik S., Andersen M.L., Moreira G.A. (2016). Phenotypic Contrasts of Duchenne Muscular Dystrophy in Women: Two Case Reports. Sleep Sci..

[5] Venugopal V., Pavlakis S. (2023). Duchenne Muscular Dystrophy. StatPearls.

[6] McGreevy J.W., Hakim C.H., McIntosh M.A., Duan D. (2015). Animal Models of Duchenne Muscular Dystrophy: From Basic Mechanisms to Gene Therapy. Dis. Model. Mech..

[7] Jin Y., Shen Y., Su X., Weintraub N.L., Tang Y. (2020). Effective Restoration of Dystrophin Expression in iPSC Mdx-Derived Muscle Progenitor Cells Using the CRISPR/Cas9 System and Homology-Directed Repair Technology. Comput. Struct. Biotechnol. J..

[8] Kodippili K., Hakim C.H., Pan X., Yang H.T., Yue Y., Zhang Y., Shin J.-H., Yang N.N., Duan D. (2018). Dual AAV Gene Therapy for Duchenne Muscular Dystrophy with a 7-Kb Mini-Dystrophin Gene in the Canine Model. Hum. Gene Ther..

[9] Duan D., Goemans N., Takeda S., Mercuri E., Aartsma-Rus A. (2021). Duchenne Muscular Dystrophy. Nat. Rev. Dis. Primer.

[10] Pedlow K., McDonough S., Lennon S., Kerr C., Bradbury I. (2019). Assisted Standing for Duchenne Muscular Dystrophy. Cochrane Database Syst. Rev..

[11] Thada P.K., Bhandari J., Umapathi K.K. (2023). Becker Muscular Dystrophy. StatPearls.

[12] Worton R.G., Burghes A.H.M., Smythies J.R., Bradley R.J. (1988). Molecular Genetics of Duchenne and Becker Muscular Dystrophy. International Review of Neurobiology.

[13] Bellayou H., Hamzi K., Rafai M.A., Karkouri M., Slassi I., Azeddoug H., Nadifi S. (2009). Duchenne and Becker Muscular Dystrophy: Contribution of a Molecular and Immunohistochemical Analysis in Diagnosis in Morocco. J. Biomed. Biotechnol..

[14] Duchenne and Becker Muscular Dystrophy. https://www.aapmr.org/about-physiatry/conditions-treatments/pediatric-rehabilitation/duchenne-and-becker-muscular-dystrophy.

[15] Romitti P.A., Zhu Y., Puzhankara S., James K.A., Nabukera S.K., Zamba G.K.D., Ciafaloni E., Cunniff C., Druschel C.M., Mathews K.D. (2015). Prevalence of Duchenne and Becker Muscular Dystrophies in the United States. Pediatrics.

[16] Gloss D., Moxley R.T., Ashwal S., Oskoui M. (2016). Practice Guideline Update Summary: Corticosteroid Treatment of Duchenne Muscular Dystrophy: Report of the Guideline Development Subcommittee of the American Academy of Neurology. Neurology.

[17] McDonald C.M., Henricson E.K., Abresch R.T., Duong T., Joyce N.C., Hu F., Clemens P.R., Hoffman E.P., Cnaan A., Gordish-Dressman H. (2018). Long-Term Effects of Glucocorticoids on Function, Quality of Life, and Survival in Patients with Duchenne Muscular Dystrophy: A Prospective Cohort Study. Lancet.

[18] Dang U.J., Damsker J.M., Guglieri M., Clemens P.R., Perlman S.J., Smith E.C., Horrocks I., Finkel R.S., Mah J.K., Deconinck N. (2024). Efficacy and Safety of Vamorolone Over 48 Weeks in Boys with Duchenne Muscular Dystrophy. Neurology.

[19] (2012). Eteplirsen. LiverTox: Clinical and Research Information on Drug-Induced Liver Injury.

[20] Wexler M. DMD Treatment: Exon-Skipping Therapies|Muscular Dystrophy News. https://musculardystrophynews.com/exon-skipping-for-duchenne-muscular-dystrophy/.

[21] Aartsma-Rus A., Corey D.R. (2020). The 10th Oligonucleotide Therapy Approved: Golodirsen for Duchenne Muscular Dystrophy. Nucleic Acid Ther..

[22] (2012). Golodirsen. LiverTox: Clinical and Research Information on Drug-Induced Liver Injury.

[23] VYONDYS 53 (Golodirsen) Injection for Patients and Caregivers|VYONDYS 53. https://www.vyondys53.com/.

[24] Viltolarsen Uses, Side Effects & Warnings. https://www.drugs.com/mtm/viltolarsen.html.

[25] How Long Does It Take for Viltepso to Work? Drugs.com. https://www.drugs.com/medical-answers/long-viltepso-work-3556811/.

[26] (2012). Viltolarsen. LiverTox: Clinical and Research Information on Drug-Induced Liver Injury.

[27] Smith C.I.E., Zain R. (2019). Therapeutic Oligonucleotides: State of the Art. Annu. Rev. Pharmacol. Toxicol..

[28] Di Fusco D., Dinallo V., Marafini I., Figliuzzi M.M., Romano B., Monteleone G. (2019). Antisense Oligonucleotide: Basic Concepts and Therapeutic Application in Inflammatory Bowel Disease. Front. Pharmacol..

[29] Haque U.S., Yokota T. (2023). Enhancing Antisense Oligonucleotide-Based Therapeutic Delivery with DG9, a Versatile Cell-Penetrating Peptide. Cells.

[30] Oberemok V.V., Laikova K.V., Repetskaya A.I., Kenyo I.M., Gorlov M.V., Kasich I.N., Krasnodubets A.M., Gal’chinsky N.V., Fomochkina I.I., Zaitsev A.S. (2018). A Half-Century History of Applications of Antisense Oligonucleotides in Medicine, Agriculture and Forestry: We Should Continue the Journey. Mol. J. Synth. Chem. Nat. Prod. Chem..

[31] Nokta M.A., Hausrath S.G., Pollard R.B. (1997). Emerging Treatments for Viral Retinitis. BioDrugs Clin. Immunother. Biopharm. Gene Ther..

[32] Perry C.M., Balfour J.A. (1999). Fomivirsen. Drugs.

[33] Fomivirsen (Intraocular Route) Side Effects—Mayo Clinic. https://www.mayoclinic.org/drugs-supplements/fomivirsen-intraocular-route/side-effects/drg-20063927?p=1.

[34] Shahryari A., Saghaeian Jazi M., Mohammadi S., Razavi Nikoo H., Nazari Z., Hosseini E.S., Burtscher I., Mowla S.J., Lickert H. (2019). Development and Clinical Translation of Approved Gene Therapy Products for Genetic Disorders. Front. Genet..

[35] Ng E.W.M., Shima D.T., Calias P., Cunningham E.T., Guyer D.R., Adamis A.P. (2006). Pegaptanib, a Targeted Anti-VEGF Aptamer for Ocular Vascular Disease. Nat. Rev. Drug Discov..

[36] Duffy A.M., Bouchier-Hayes D.J., Harmey J.H. (2013). Vascular Endothelial Growth Factor (VEGF) and Its Role in Non-Endothelial Cells: Autocrine Signalling by VEGF. Madame Curie Bioscience Database.

[37] Pegaptanib (Intraocular Route) Side Effects—Mayo Clinic. https://www.mayoclinic.org/drugs-supplements/pegaptanib-intraocular-route/side-effects/drg-20070848.

[38] Wong E., Goldberg T. (2014). Mipomersen (Kynamro). Pharm. Ther..

[39] Hashemi N., Odze R.D., McGowan M.P., Santos R.D., Stroes E.S.G., Cohen D.E. (2014). Liver Histology During Mipomersen Therapy for Severe Hypercholesterolemia. J. Clin. Lipidol..

[40] Stein E.A., Dufour R., Gagne C., Gaudet D., East C., Donovan J.M., Chin W., Tribble D.L., McGowan M. (2012). Apolipoprotein B Synthesis Inhibition with Mipomersen in Heterozygous Familial Hypercholesterolemia: Results of a Randomized, Double-Blind, Placebo-Controlled Trial to Assess Efficacy and Safety as Add-on Therapy in Patients with Coronary Artery Disease. Circulation.

[41] Nusinersen (Spinraza®)—Spinal Muscular Atrophy (SMA)|National Institute of Neurological Disorders and Stroke. https://www.ninds.nih.gov/about-ninds/what-we-do/impact/ninds-contributions-approved-therapies/nusinersen-spinrazar-spinal-muscular-atrophy-sma.

[42] SPINRAZA® (Nusinersen) Safety & Side Effects. https://www.spinraza.com/en_us/home/why-spinraza/safety-profile.html.

[43] Defitelio (Defibrotide Sodium). *FDA* 2019. https://www.fda.gov/drugs/resources-information-approved-drugs/defitelio-defibrotide-sodium.

[44] Defitelio® (Defibrotide Sodium)|Pathophysiology of VOD/SOS. https://defitelio.com/vod/pathophysiology-of-vod.

[45] Defibrotide. https://go.drugbank.com/drugs/DB04932.

[46] Drug Trial Snapshot: TEGSEDI. *FDA* 2021. https://www.fda.gov/drugs/drug-approvals-and-databases/drug-trial-snapshot-tegsedi.

[47] What Is Transthyretin Amyloidosis (ATTR Amyloidosis): Symptoms, Diagnosis & Treatment|Pfizer. https://www.pfizer.com/disease-and-conditions/attr-amyloidosis.

[48] Transthyretin Amyloidosis (ATTR-CM): Types, Causes, Treatment. Cleveland Clinic. https://my.clevelandclinic.org/health/diseases/17855-amyloidosis-attr.

[49] Bitan G., Teplow D.B. (2019). Chapter Fifteen—Disease-Modifying Therapy for Proteinopathies: Can the Exception Become the Rule?. Progress in Molecular Biology and Translational Science.

[50] Burel S.A., Hart C.E., Cauntay P., Hsiao J., Machemer T., Katz M., Watt A., Bui H.-H., Younis H., Sabripour M. (2016). Hepatotoxicity of High Affinity Gapmer Antisense Oligonucleotides Is Mediated by RNase H1 Dependent Promiscuous Reduction of Very Long Pre-mRNA Transcripts. Nucleic Acids Res..

[51] Swayze E.E., Siwkowski A.M., Wancewicz E.V., Migawa M.T., Wyrzykiewicz T.K., Hung G., Monia B.P., Bennett C.F. (2007). Antisense Oligonucleotides Containing Locked Nucleic Acid Improve Potency but Cause Significant Hepatotoxicity in Animals. Nucleic Acids Res..

[52] McClorey G., Banerjee S. (2018). Cell-Penetrating Peptides to Enhance Delivery of Oligonucleotide-Based Therapeutics. Biomedicines.

[53] Kamola P.J., Maratou K., Wilson P.A., Rush K., Mullaney T., McKevitt T., Evans P., Ridings J., Chowdhury P., Roulois A. (2017). Strategies for In Vivo Screening and Mitigation of Hepatotoxicity Associated with Antisense Drugs. Mol. Ther. Nucleic Acids.

[54] Amondys 45 (Casimersen) Injection, for Intravenous Use: US Prescribing Information. https://www.accessdata.fda.gov/drugsatfda_docs/label/2021/213026lbl.pdf.

[55] Iannaccone S., Phan H., Straub V., Muntoni F., Wolf D., Malhotra J., Chu R., Darton E., Mercuri E. (2022). Casimersen in Patients with Duchenne Muscular Dystrophy Amenable to Exon 45 Skipping: Interim Results from the Phase 3 ESSENCE Trial. Neuromuscul. Disord..

[56] Wagner K.R., Kuntz N.L., Koenig E., East L., Upadhyay S., Han B., Shieh P.B. (2021). Safety, Tolerability, and Pharmacokinetics of Casimersen in Patients with Duchenne Muscular Dystrophy Amenable to Exon 45 Skipping: A Randomized, Double-blind, Placebo-controlled, Dose-titration Trial. Muscle Nerve.

[57] Carver M.P., Charleston J.S., Shanks C., Zhang J., Mense M., Sharma A.K., Kaur H., Sazani P. (2016). Toxicological Characterization of Exon Skipping Phosphorodiamidate Morpholino Oligomers (PMOs) in Non-Human Primates. J. Neuromuscul. Dis..

[58] Sarepta Therapeutics Announces FDA Approval of AMONDYS 45TM (Casimersen) Injection for the Treatment of Duchenne Muscular Dystrophy (DMD) in Patients Amenable to Skipping Exon 45|Sarepta Therapeutics, Inc. https://investorrelations.sarepta.com/news-releases/news-release-details/sarepta-therapeutics-announces-fda-approval-amondys-45tm.

[59] (2012). Casimersen. LiverTox: Clinical and Research Information on Drug-Induced Liver Injury.

[60] Sarepta Therapeutics, Inc. (2021). A Randomized, Double-Blind, Placebo-Controlled, Dose-Titration, Safety, Tolerability, and Pharmacokinetics Study Followed by an Open-Label Safety and Efficacy Evaluation of SRP-4045 in Advanced-Stage Patients with Duchenne Muscular Dystrophy Amenable to Exon 45 Skipping; Clinical Trial Registration NCT02530905; clinicaltrials.gov. NCT02530905.

[61] Marta Figueiredo FDA Approves Amondys 45 for DMD Patients Amenable to Exon 45 Skipping. Muscular Dystrophy News. https://musculardystrophynews.com/news/fda-approves-amondys-45-casimersen-sarepta-duchenne-exon-45-skipping-therapy/.

[62] Alhamadani F., Zhang K., Parikh R., Wu H., Rasmussen T.P., Bahal R., Zhong X.-B., Manautou J.E. (2022). Adverse Drug Reactions and Toxicity of the Food and Drug Administration-Approved Antisense Oligonucleotide Drugs. Drug Metab. Dispos. Biol. Fate Chem..

